# Patient-Centered Education Through A Massive Open Online Course (MOOC) for Patients With Multiple Myeloma and Caregivers: Descriptive Study of Knowledge Gains by French Association of Patients With Multiple Myeloma (AF3M) and French-Speaking Myeloma Intergroup (IFM)

**DOI:** 10.2196/81225

**Published:** 2026-02-25

**Authors:** Alexis Talbot, Bernard Delcour, Laurent Gillot, Bertrand Arnulf, Hervé Avet Loiseau, Catherine Boccaccio, Laurent Frenzel, Lionel Karlin, Karen Kraeuter, Margaret Macro, Mohamad Mohty, Aurore Perrot, Cyrille Touzeau, Cécile Sonntag, Cyrille Hulin, Philippe Moreau, Olivier Decaux

**Affiliations:** 1 UCSF Helen Diller Family Comprehensive Cancer Center, Parnassus Avenue, San Francisco, CA, United States, 1 4156239672; 2 French Association of Patients with Multiple Myeloma (AF3M), Paris, France; 3 Hopital Saint Louis, Assistance Publique Hôpitaux de Paris (APHP), Paris, France; 4 Unite de Genomique du Myélome, Institut National de la Santé et de la Recherche Medicale, University Cancer Center of Toulouse, Toulouse, France; 5 Intergroupe Francophone du Myélome, Paris, France; 6 Hopital Necker, Assistance Publique Hôpitaux de Paris (APHP), Paris, France; 7 Centre Hospitalier Lyon Sud, Lyon, France; 8 Onco-psychologist, Bordeaux, France; 9 Hematology department, CHU CAEN, Caen, France; 10 Hematology department, Hopital Saint Antoine, Assistance Publique Hôpitaux de Paris (APHP), Paris, France; 11 Université de Toulouse, CHU de Toulouse, Hématologie, IUCT-Oncopole CRCT, Toulouse, France; 12 CHU de Nantes, Hôtel Dieu, Nantes, France; 13 CHRU de Strasbourg, Hôpital Hautepierre, Strasbourg, France; 14 Hematology department, CHU Bordeaux, Bordeaux, France; 15 Hematology department, CHRU Hôpital de Pontchaillou, Rennes, France

**Keywords:** MOOC, multiple myeloma, patient, medical education, therapeutic patient education, massive open online course

## Abstract

**Background:**

Multiple myeloma (MM) is a chronic hematologic malignancy characterized by complex therapeutic strategies, repeated relapses, and substantial information and psychosocial needs. Advances in oral therapies and outpatient management have shifted greater responsibility to patients and caregivers, emphasizing the need for accessible, high-quality educational resources. Therapeutic patient education (TPE) aims to empower patients to understand and manage their condition more effectively. Digital education tools such as massive open online courses (MOOCs) represent an innovative approach to deliver structured, interactive, and scalable learning experiences to large patient populations. However, few MOOCs have been specifically designed for patients with oncological or hematological disorders, and even fewer have been rigorously evaluated for their educational impact.

**Objective:**

This study aimed to develop and evaluate a MOOC co-designed with patients, caregivers, and health care professionals to improve knowledge, skills, and empowerment among patients living with MM and their relatives. Secondary objectives included assessing participant satisfaction, engagement, and the feasibility of this digital education model at a national scale.

**Methods:**

The MOOC “Understanding and Living with Myeloma” was jointly developed by the French Association of Patients with Multiple Myeloma (AF3M) and the French-Speaking Myeloma Intergroup (IFM). The program consisted of 5 thematic modules delivered over 8 weeks, covering disease mechanisms, diagnosis, treatment options, side-effect management, and daily-life adaptation. Content combined educational videos, self-assessment quizzes, peer-tutoring forums, and live web conferences with experts. Participants self-assessed their knowledge using a 52-item questionnaire rated from 1 (poor) to 5 (excellent) before and after completing the program. Descriptive and inferential analyses were performed using the Wilcoxon signed-rank test (2-sided *α*=.05).

**Results:**

During the first session, 254 participants registered for the course. Among them, 76 (30%) completed all modules and both evaluations. The mean global knowledge score increased from 3.06/5 before to 4.21/5 after the MOOC (mean gain + 1.15, + 38%; *P*<.001). Improvements were consistent across all knowledge domains, including understanding of treatments (+40%), recognition of warning signs (+35%), and self-management skills (+39%). Overall, 98% (74/76) of respondents reported being satisfied or very satisfied with the course, and 99% (75/76) would recommend it to other patients. Since 2018, the MOOC has been conducted 6 times at different periods, enrolling a cumulative total of 2400 participants, confirming its sustainability and scalability.

**Conclusions:**

Participation in this co-designed, patient-centered MOOC was associated with a statistically and educationally significant improvement in knowledge among patients with MM and their caregivers. The program was highly valued by users and demonstrates the feasibility of large-scale digital patient education in oncology. As a freely accessible, repeatable, and peer-supported resource, this MOOC complements medical consultations and traditional TPE programs. Its design and outcomes may serve as a model for future digital health education initiatives targeting other chronic diseases.

## Introduction

Cancer is now often regarded as a chronic condition. Today, more than 3 million people in France are living with or after a cancer diagnosis. Within the objective of preserving quality of life, education and information for patients play a central role. Health professionals are now expected to engage in educational activities that empower patients while acknowledging the various barriers they face at different stages of illness. Prevention and therapeutic education strategies are crucial to limit the negative impact of disease and treatment on patients’ daily lives and to support their social and professional reintegration. A major challenge in modern oncology is thus the implementation of educational programs that help patients cope better with treatment and its associated difficulties [1]. Multiple myeloma (MM) accounts for approximately 10%‐15% of blood cancers. Despite therapeutic progress leading to improved survival, MM remains incurable and is marked by successive relapses. Recent advances have shifted care toward outpatient and home-based treatment, often involving oral therapies. This evolution highlights the need for new, patient-centered communication strategies. Unfortunately, the growing number of patients and limited consultation time often prevent health care professionals from fully engaging in their educational mission within hospital settings.

Therapeutic patient education (TPE) draws on medical science, education, and the social sciences [2]. It encourages patients to become active participants in their care. According to the World Health Organization, TPE aims to help patients acquire or maintain skills necessary to better manage life with a chronic illness. It is a personalized, multidisciplinary approach involving both health care providers and patient associations. In this context, digital tools such as massive open online courses (MOOCs) offer an innovative solution. A MOOC is a free, open-access online course that combines video content, quizzes, self-assessments, and discussion forums to encourage both learning and interaction. MOOCs are primarily used for training students and health care professionals; few have been designed for patient education [3-5]. Learners can proceed at their own pace, interact with peers and instructors, and revisit content as needed, features that are particularly well-suited for patients living with complex diseases, but few are designed to improve understanding of diagnostic processes [6] or to prepare patients for surgery [7]. Originally developed by North American academic institutions, MOOCs are now recognized for their potential in medical education, especially among patients with chronic conditions, by improving health literacy, self-management, and engagement in their care. Recent studies have demonstrated the feasibility and positive impact of digital self-management and educational interventions on chronic conditions; for example, the MyRelief program showed that an online educational platform could effectively support self-management and improve outcomes in patients with persistent low back pain [8]. The importance of user-centered and co-designed digital education tools has also been highlighted in other chronic disease contexts; for instance, integrating lived experience and expert input in the development of a digital stroke prevention platform enhanced its usability, engagement, and potential for behavior change [9]. In the field of education for patients with oncological disorders, Álvarez-Pérez et al [10] demonstrated that co-creating a MOOC with patients and professionals can effectively enhance digital health literacy and promote person-centered care among women living with breast cancer, underscoring the value of participatory design in digital health interventions. The potential of MOOCs as accessible educational interventions for chronic musculoskeletal conditions has been further explored in the MOOC-osteoarthritis randomized controlled trial, which aims to evaluate whether a MOOC can improve disease-specific knowledge and pain self-efficacy among patients with hip and/or knee osteoarthritis [11]. In oncology, MOOCs have also been successfully used as professional training tools, like a nationwide French MOOC on geriatric oncology that effectively disseminated specialized knowledge and fostered interdisciplinary collaboration among health care professionals involved in the care of older patients with cancer [12].

Despite the availability of information, many patients report insufficient understanding of the disease, its mechanisms, treatment side effects, and patient rights. Traditional TPE programs require small-group settings and significant time investment and have not been consistently implemented across all regions. The French Association of Patients with Multiple Myeloma (AF3M) has shown a strong commitment to developing innovative educational solutions. To our knowledge, no MOOC had previously been developed for patients with MM. The main objective of our work was therefore to create and implement a MOOC specifically for such patients and their relatives. The goal was to help participants acquire knowledge, skills, and abilities to improve disease self-management and quality of life. The impact of this educational tool was assessed using a pre- and postcourse self-evaluation of knowledge.

## Methods

### Overview

The MOOC “Understanding and Living with Myeloma” was developed by the AF3M, a nonprofit organization founded in 2007 by 27 patients and relatives. As of today, this association includes more than 2000 members and over 100 volunteers and has held ministerial recognition since 2012. Its missions include providing information and support to patients and caregivers, advocating for patient rights, promoting public awareness of MM, supporting research, and facilitating therapeutic education initiatives across France. To promote access to reliable information and empower patients to better manage their illness, the AF3M made the decision to innovate through digital education. In response to the growing relevance of online learning, it initiated the development of this MOOC dedicated specifically to patients. The French-Speaking Myeloma Intergroup (IFM), a cooperative clinical and research network created in 1994, partnered with the AF3M on this project. The IFM includes hematologists, clinicians, and biologists in France and has been instrumental in conducting large-scale clinical trials and advancing both basic and translational myeloma research.

Expertise in pedagogical engineering, audiovisual production, and digital learning design was provided by HB Motion, an educational media company. The project received institutional support from the Janssen laboratory (Johnson & Johnson Group). A steering committee was established, initially composed of patients and caregivers, and subsequently expanded to include members of the AF3M’s scientific board, comprising approximately 30 health care professionals affiliated with the IFM. The final project team consisted of a dozen AF3M volunteers, patients, relatives, and multimedia learning engineers, who worked in close collaboration to create content tailored to patient needs (Textbox 1). Participants were recruited between September 2018 and October 2018 through the AF3M networks. Announcements were systematically disseminated via AF3M’s website, newsletters, hospital hematology departments, and social media. No exclusion criteria were applied other than language proficiency in French and internet access. Enrollment was open to all interested participants via online registration. Recruitment was therefore open and national in scope, ensuring representation from various French regions. Participation in the MOOC was also open to caregivers and health professionals wishing to deepen their understanding of the disease, therapeutic approaches, and patient-centered challenges. The MOOC was officially launched during the National Myeloma Information Day in 2018. A self-assessment questionnaire at the start of the MOOC had to be completed online. The questions were about initial knowledge and skills. The same questionnaire was administered again at the end of the MOOC to participants who completed the entire program. The pre-MOOC and post-MOOC self-assessments of the 2018 edition were analyzed and compared.

Textbox 1. **Goals of the massive open online course (MOOC) concerning knowledge, skills, and capacities.**

**Improve knowledge**

- Understand what multiple myeloma is

- Know the treatments for multiple myeloma

- Know the side effects of the treatments

**Increase know-how**

- Knowing how to interpret results

- Know how to prepare your consultation with the hematologist

- Use tips and tricks to improve your daily life

- Learn to better manage your pain and fatigue during and after treatments

- Help patients to control their illness and its consequences

- Seek help for the sick as well as for the “helpers”

**Reinforce skills**

- Be more comfortable with your illness and the consequences on daily life

- Be more comfortable in a consultation

- Communicate better with loved ones

- Be an attentive caregiver

**After attending a therapeutic education program, the person may be able to**

- Better understand their illness and thus be able to accept it

- Know the preventive measures to be adopted: environmental planning, taking preventive treatment

- Recognize an aggravation and know how to react adequately

- Identify the factors or circumstances triggering peaks of resurgence of the disease to better avoid them

- Overcome daily difficulties linked to the disease (improve everyday life)

All statistical analyses were conducted using Bioconductor packages in the R programming language (R Core Team). The unpaired Wilcoxon test was used to compare pre- and posttest continuous variables, and Fisher exact test was used for categorical variables. All statistical tests were 2-sided, and *P* values less than or equal to .05 were considered statistically significant.

### Ethical Considerations

This study did not involve any biomedical experimentation or collection of identifiable personal data. Under French law, only research involving the human person (RIPH) requires prior review by an Institutional Review Board. RIPH is legally defined as research that is organized and conducted on persons with the aim of developing biological or medical knowledge (French Public Health Code Article R1121-1) [13]. This study consisted of the evaluation of an educational program developed for patients with MM and their caregivers by the AF3M in collaboration with the IFM using anonymized self-assessment questionnaires and did not entail any biomedical intervention, randomization, or modification of usual care. It therefore falls outside the scope of RIPH and did not require the Ethics Committee for the protection of persons review under the applicable framework. National guidance issued by the French National Agency for Medicines and Health Products Safety (ANSM) confirms that such noninterventional educational evaluations are exempt from ethics committees for the protection of persons’ authorization. Data were anonymized before analysis, and data processing complied with the European General Data Protection Regulation and the French Data Protection Act. Participation was voluntary, and all users provided electronic informed consent when registering on the MOOC platform.

## Results

### Participant Engagement and Expectations

Following the survey sent by email to all AF3M members, 398 responses were obtained from 344 patients with MM (86%), 47 relatives (12%), and 7 (2%) health professionals. The average age of patients with MM was 64.8 years (range 32‐86), with 54% being male. Only 10 patients (3%) had participated previously in TPE programs. Responses were geographically distributed across the country. The median follow-up of patients with MM was 5 years. Respondents expressed their expectations regarding knowledge, skills, and attitudes to be developed to improve the management of the disease. In total, 68% (n=270) of respondents said they were interested in the MOOC project. The classification of attitudes that patients wished to develop was optimism (71.9%), resilience (62.7%), fighting spirit (57%), self-confidence (56%), autonomy (55%), courage (46%), attention to relatives (46%), perseverance (39%), attention to medical prescriptions (36%), listening skills (35%), communication (34%), self-control (33%), adaptability (27%), and organization (17%).

### MOOC Development and Structure

The educational path includes 5 modules that are divided into 15 stages over a period of 8 weeks for each session of the MOOC (Textbox 2). All modules contain short videos, patient testimonials, interviews with health professionals, and analyses conducted from real situations. The modules end with a challenge, a summary, a quiz, feedback on the proposed activities, an assessment of the skills acquired, and a validation badge. At the end of each module, participants can attend a thematic web conference, led by a health care professional and an AF3M representative, where they can ask questions by webchat. These exchanges are able to be replayed. The interactive MOOC is tutored by peers and AF3M volunteers. From the second session, the tutors are accompanied and supported by an onco-psychologist to help enable them to handle any emergency situations. When registering for the MOOC, participants complete a questionnaire on demographic characteristics (Table 1).

Textbox 2. **Schedule of the massive open online course (MOOC)**

Module 1: Introduction: Presentation of the MOOC and instructions for use

Module 2: What is multiple myeloma?

Module 3: Treatments for multiple myeloma

Module 4: Living better with multiple myeloma

Module 5: How to manage relationships with those around you?

Conclusion

**Table 1.** Characteristics of massive open online course (MOOC) participants (N=288).

Characteristics	Values, n (%)
Sex	
Female	118 (41)
Male	170 (59)
Time from diagnosis (year)	
<1	37 (13)
≥1-5	121 (42)
≥5-10	58 (20)
≥10	14 (5)
Missing	58 (20)
Type of participant	
Patient	181 (63)
Caregivers	75 (26)
Health care professional	23 (8)
Other	9 (3)
Age (years)	
<40	17 (6)
41–50	40 (14)
51–60	78 (27)
61–70	95 (33)
71–80	49 (17)
>80	9 (3)
Status	
Active worker	78 (27)
Medical leave	35 (12)
Invalidity	30 (7)
Retired	138 (48)
Other	17 (6)

### Participation and Satisfaction Rate

**Figure 1.**
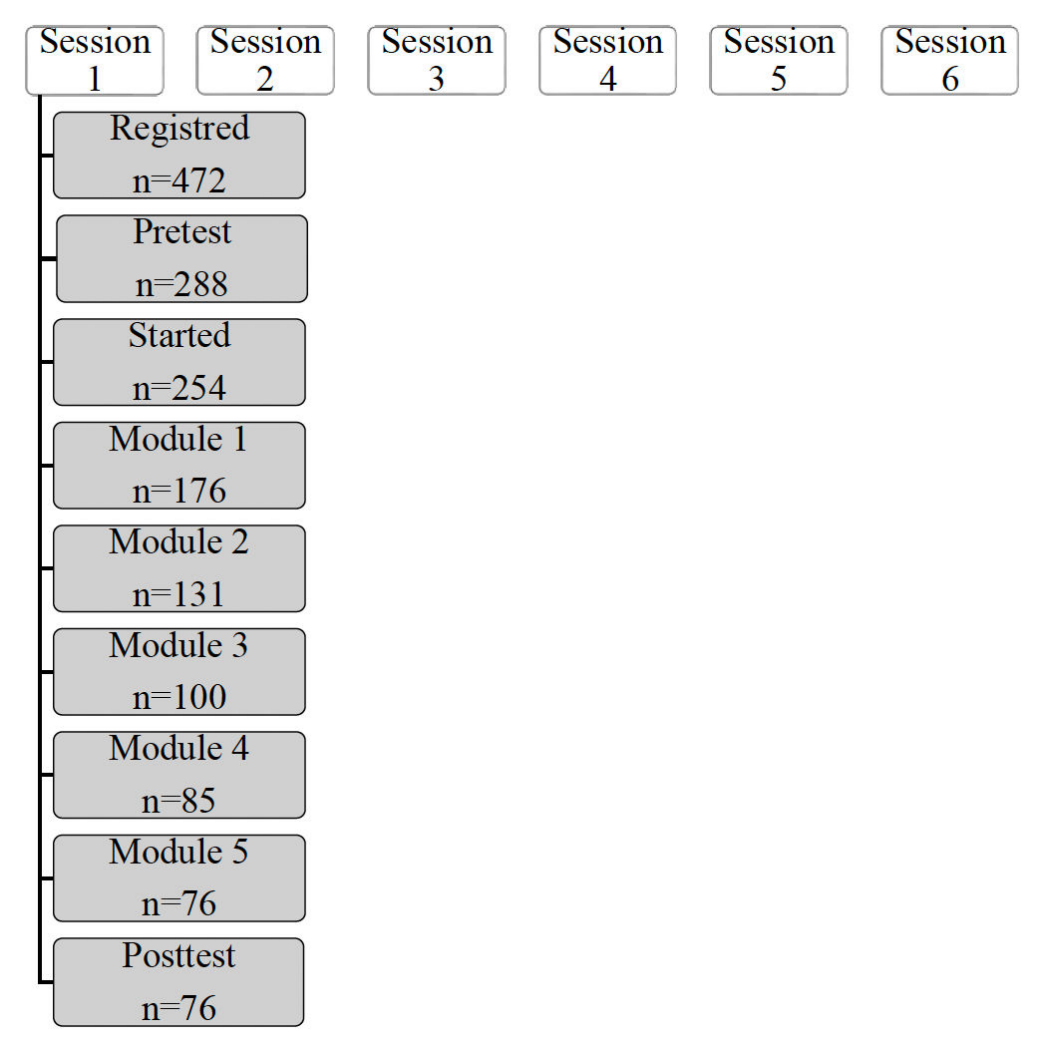
Flowchart of massive open online course (MOOC) participation.

At the first edition of the MOOC, 30% of participants (76/254) completed all modules (Figure 1), and 98% of participants rated this training as satisfactory or very satisfactory, and 99% would recommend this MOOC to other people. The results regarding the satisfaction rates are summarized in Table 2. The different sessions gave rise to many exchanges between the participants and the volunteer tutors responsible for responding to and encouraging them. In total, more than 7500 contributions (questions and messages) have been published. This MOOC has been conducted 6 times at different periods, with a cumulative enrollment of 2400 participants. This knowledge assessment was conducted only during the first session.

**Table 2.** Results regarding the rates of satisfaction.

Questions	Responses, n (%)
Overall assessment of the MOOC^a^	
Very satisfied	50 (70)
Satisfied	17 (24)
Moderately satisfied	3 (4)
Not very satisfied	2 (2)
Would you recommend this MOOC to a loved one or a patient?	
Yes	71 (99)
No	1 (1)
How useful was this session?	
Very useful	54 (75)
Useful	17 (24)
Not very useful	1 (1)

a MOOC: massive open online course.

### Knowledge: Pretest and Posttest Evaluations

**Figure 2.**
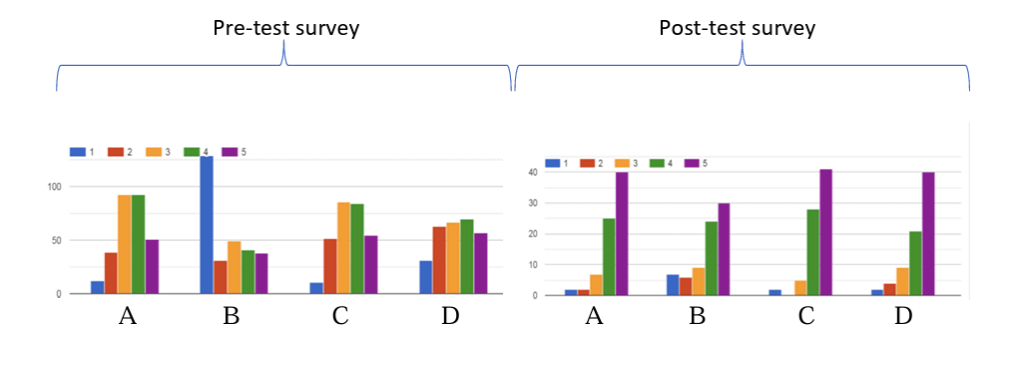
Patients' response to pre- and posttest surveys (number of responses, level of knowledge: 1=poor; 2=fair; 3=good; 4=very good; 5=excellent). A. What is a multiple myeloma disease? B. What is a monoclonal gammopathy of undetermined significance? C. What are the symptoms of multiple myeloma disease? D. What are the risks of relapse?

General knowledge of MM, warning signs, treatments, know-how, and skills (52 items in total) was assessed by the patients themselves before starting the MOOC (n=288) and at the end of the training (n=76) by scoring items from 1 to 5 (5 representing excellent knowledge) (Figure 2). Mean knowledge scores improved from 3.06/5 pretest to 4.21/5 posttest (gain of +1.15, 38%; *P*<.001) (Table 3).

**Table 3.** Results of the pre- and posttest surveys and change.

Item to be scored using scale of 1 to 5 (5 represents excellent) of the knowledge	Before MOOC^a^ (n=288), mean (SD)	After MOOC (n=76), mean (SD)	Wilcoxon test, mean difference (%)	*P* value
General knowledge of multiple myeloma				
What is multiple myeloma	3.46 (1.06)	4.30 (0.94)	+0.84 (+24%)	.045
What is a monoclonal gammopathy of undetermined significance	2.40 (1.49)	3.84 (1.29)	+1.44 (+60%)	.03
Symptoms of the disease	3.42 (1.1)	4.39 (0.83)	+0.97 (+28%)	.01
Risks of relapse	3.20 (1.28)	4.22 (1.03)	+1.02 (+32%)	.03
The role of white blood cells	3.25 (1.2)	4.22 (0.97)	+0.97 (+30%)	.01
The mechanisms of multiple myeloma	2.85 (1.18)	4.03 (1.1)	+1.18 (+41%)	.008
The monoclonal peak	3.08 (1.28)	4.13 (1.06)	+1.05 (+34%)	.04
Clinical and biological monitoring of the disease	3.01 (1.21)	4.03 (1.08)	+1.02 (+34%)	.01
Knowledge of the warning signs linked to multiple myeloma and its treatments				
Pain	3.32 (1.26)	4.34 (0.89)	+1.02 (+31%)	.01
The risk of thrombosis	2.73 (1.33)	3.96 (0.96)	+1.23 (+45%)	.02
Beginning of infection	3.00 (1.33)	4.12 (0.97)	+1.12 (+37%)	.01
Neuropathy	2.99 (1.43)	4.28 (0.96)	+1.29 (+43%)	.04
Side effects of drugs	3.06 (1.29)	4.22 (0.89)	+1.16 (+38%)	.046
Name of medication	3.40 (1.46)	4.14 (1.19)	+0.74 (+22%)	.02
Know the therapeutic objective of each drug	2.80 (1.35)	3.78 (1.15)	+0.98 (+35%)	.04
Identify myeloma-specific drugs from other drugs	3.04 (1.46)	4.07 (1.11)	+1.03 (+34%)	.005
Know the dosage of treatments and when to take them	3.24 (1.52)	4.25 (1.18)	+1.01 (+31%)	.007
Understand the discontinuous rhythm of treatment	2.85 (1.41)	4.12 (1.12)	+1.27 (+45%)	.03
Describe the potential side effects of treatment	3.09 (1.32)	4.20 (1.08)	+1.11 (+36%)	.04
Know the possible impact of treatment on sex life	2.69 (1.4)	4.05 (1.11)	+1.36 (+51%)	.03
Know the effects of treatment on fertility and the teratogenicity	2.81 (1.46)	4.04 (1.2)	+1.23 (+44%)	.04
Understand treatment with autograft	3.14 (1.44)	4.32 (1.11)	+1.18 (+38%)	.01
Differentiate between autograft and allograft	3.49 (1.53)	4.43 (1.02)	+0.94 (+27%)	.01
Other knowledge				
Social rights	2.33 (1.19)	3.83 (1.09)	+1.50 (+64%)	.006
The value of physical activity	3.40 (1.28)	4.66 (0.78)	+1.26 (+37%)	.03
The benefits of a balanced diet	3.49 (1.18)	4.58 (0.88)	+1.09 (+31%)	.02
The advantage of practicing an adapted physical activity	3.36 (1.24)	4.61 (0.87)	+1.25 (+37%)	.01
External aid	2.61 (1.25)	4.08 (1.02)	+1.47 (+56%)	.01
Know how to				
Detect onset of fever	3.10 (1.34)	4.28 (0.93)	+1.18 (+38%)	.01
Persistent pain	3.08 (1.3)	4.28 (0.92)	+1.20 (+39%)	.01
Fatigue	3.14 (1.27)	4.42 (0.9)	+1.28 (+41%)	.04
Signs of thrombosis	2.58 (1.38)	3.89 (1.05)	+1.31 (+51%)	.007
Persistent infectious signs	2.96 (1.37)	4.21 (1.05)	+1.25 (+42%)	.02
Signs of neuropathy	2.82 (1.39)	4.36 (0.93)	+1.54 (+55%)	.045
Appearance of side effects of treatments	2.86 (1.44)	4.11 (0.88)	+1.25 (+44%)	.03
Manage medication administration calendar	3.72 (1.46)	4.63 (0.91)	+0.91 (+25%)	.048
Manage the stock of medicines	3.73 (1.48)	4.61 (0.91)	+0.88 (+24%)	.001
Plan how to take treatment	3.58 (1.53)	4.58 (1.12)	+1.00 (+28%)	.02
Know what to do if you forget to take treatment	3.04 (1.45)	4.26 (0.9)	+1.22 (+40%)	.048
Identify how to take treatment regularly	3.56 (1.41)	4.51 (1.07)	+0.95 (+27%)	.02
Know what to do in case of side effects of treatment	2.86 (1.58)	4.05 (1.58)	+1.19 (+42%)	.01
Self-inject drugs subcutaneously	2.32 (1.41)	3.28 (1.31)	+0.96 (+41%)	.02
Know how to self-medicate	2.43 (1.26)	3.51 (0.94)	+1.08 (+44%)	.03
Skills				
Communicate with caregivers about the disease	3.24 (1.2)	4.29 (1.02)	+1.05 (+32%)	.04
Explain the illness and the treatments to family and friends	3.16 (1.31)	4.33 (0.86)	+1.17 (+37%)	.01
Alert in case of adverse effects of the treatment	3.21 (1.35)	4.34 (0.94)	+1.13 (+35%)	.03
Know how to call in case of warning signs or worsening	3.18 (1.33)	4.41 (0.93)	+1.23 (+39%)	.02
Know how to ask for help	2.98 (1.32)	4.33 (0.93)	+1.35 (+45%)	.03
Discuss difficulties with the medical team	3.11 (1.3)	4.34 (0.95)	+1.23 (+40%)	.045
Express difficulties with family or a caregiver	2.99 (1.39)	4.22 (0.93)	+1.23 (+41%)	.02
Know who and how to call in case of alert or aggravation	3.13 (1.39)	4.34 (1.14)	+1.21 (+39%)	.02
Share experience of the disease with someone	2.91 (1.33)	4.17 (1.07)	+1.26 (+43%)	.03
All items	3.06 (0.33)	4.21 (0.27)	+1.15 (38%)	<.001

a MOOC: massive open online course.

### Web Conference Impact

The interactive online conferences brought together an average of 100 participants and are widely consulted in replay. An expert is interviewed by a mediator for one hour. During the COVID-19 pandemic, which further exacerbated existing anxiety and concerns among patients with MM, the AF3M provided help and support to them and their families during this exceptional health crisis. In response, the AF3M hosted web conferences on COVID-19. In total, 600 people registered for the first web conference, which was substantially more than the expected amount (Table 4). This outcome confirmed the importance of opening a virtual space for patients with hemopathies to ask questions and obtain answers from experts and from cooperative groups. The level of satisfaction rose to 98% by the third web conference. These web conferences delivered clear information, enhanced knowledge, enabled interaction with experts, and addressed COVID-19 from medical, psychological, legal, and social perspectives.

**Table 4.** Web conference data during the COVID-19 crisis.

Conference	1	2	3	4
Number of registrants	590	603	636	330
Number of participants	473	466	485	262
Questions raised before the web conference	49	36	24	15
Number of questions asked through the chat	—^a^	230	182	51
Satisfaction rate (%)	68	84	98	88
Speaker 1	Infectious disease specialist	Hematologist	Hematologist	Hematologist
Speaker 2	Hematologist	Hematologist	Hematologist	None
Speaker 3	None	Onco-psychologist	Onco-psychologist	Onco-psychologist
Speaker 4	None	Health lawyer	Health lawyer	None

a Not applicable.

## Discussion

### Principal Findings

This MOOC represents one of the first educational platforms specifically designed to support patients with MM and their relatives. Completion of the MOOC led to a substantial improvement in participants’ understanding of MM, including disease mechanisms, warning signs, available treatments, and self-management skills. The mean knowledge score rose from 3.06 to 4.21 out of 5, a+38% increase (*P*<.001), confirming the educational effectiveness of the program.

### Interpretation and Comparison With Existing Literature

Developed in collaboration with patients and health care professionals, it addresses key needs, including access to scientifically validated information, support in coping with treatment-related challenges, and the opportunity to share experiences within a safe, anonymous, and interactive environment. This initiative offers important advantages. It provides free and accessible information to patients across all geographic areas, including those who may be isolated or far from specialist centers. The platform fosters open peer-to-peer and patient-expert discussions. The inclusion of web conferences and replays further enhances the value of the program, allowing patients to engage in real time or at their convenience. Additional strengths are the availability of web conferences, interactive exchanges, and tutoring. The overwhelmingly positive feedback and high satisfaction rates observed across four conference editions demonstrate both the relevance and the acceptability of this digital educational tool. Our findings align with and expand upon previous work demonstrating the value of digital and co-designed educational interventions for patients with chronic diseases [7]. Similar knowledge gains were observed in oncology-focused MOOCs, such as the program described for women with breast cancer, where a participatory design approach improved digital health literacy and engagement [10]. Comparable improvements in knowledge (25%‐40%) have been reported in other chronic conditions, including the MOOC-osteoarthritis trial for osteoarthritis and the MyRelief self-management platform for persistent low back pain [8]. These results collectively suggest that structured, user-centered digital education can promote understanding, self-efficacy, and behavioral adaptation across diverse patient populations.

In contrast to most previously published MOOCs, which primarily targeted health care professionals or single disease educational objectives, our study uniquely focused on patients with a specific hematologic malignancy and actively involved caregivers and advocacy groups from the outset. This co-construction process with AF3M and IFM ensured content relevance, scientific accuracy, and a strong sense of community ownership. The 38% relative improvement in knowledge and very high satisfaction rate (98%) are therefore consistent with the literature while confirming the added value of participatory design and peer tutoring in enhancing engagement.

Furthermore, our results support evidence that interactive components, such as web conferences, discussion forums, and tutor guidance, substantially increase learning outcomes and emotional support compared with passive e-learning formats. These findings reinforce earlier observations from geriatric oncology and bariatric surgery MOOCs, in which interactivity and peer exchange were key determinants of user satisfaction. Together, these comparisons highlight that the present MOOC not only replicates but also extends existing evidence by demonstrating that co-designed digital education can be successfully implemented and sustained in a complex oncologic setting.

### Limitations

Nevertheless, several limitations must be acknowledged. First, the digital nature of the program poses challenges for some patients. Barriers include limited access to computers or mobile devices, unfamiliarity with digital tools, or language limitations, as the MOOC is only available in French. This is particularly relevant in MM, a disease that predominantly affects older patients, some of whom may also experience physical or cognitive impairments (neuropathy, fatigue, or pain) that can interfere with concentration and learning. While basic computer literacy is required, the presence of peer tutors and access to technical tutorials aim to mitigate these barriers. Second, the potential for selection bias must be considered. Participants were largely recruited through the AF3M’s network, likely including patients already motivated to learn more about their condition. In addition, self-assessment questionnaires, while informative, are subjective in nature and were developed by the team involved in designing the MOOC, raising concerns about possible measurement bias [14]. Although more objective outcome measures (quality of life and adherence) would strengthen the evaluation, such endpoints typically require a clinical trial framework [14]. As commonly observed in MOOC-based learning, we noted a progressive decrease in participant engagement over time. Only 30% of learners completed the full program.

Interestingly, the 10 hours of structured patient education provided by the MOOC may, indirectly, allow clinicians to focus on more personalized and specific aspects of care during consultations. In this way, the MOOC complements rather than replaces the essential in-person interactions with medical and paramedical teams. Beyond knowledge acquisition, this program may also support emotional well-being, enhance patient autonomy, and foster a sense of community among participants. The platform also proved highly adaptable, offering timely content during the COVID-19 pandemic, when patients’ anxiety was heightened and access to care was disrupted. This success of the web conferences underlined the critical importance of offering a virtual space for reliable, empathetic, and timely medical communication.

### Broader Implications and Conclusions

MOOCs represent a flexible and scalable solution for therapeutic education, allowing for regular updates as scientific knowledge evolves and offering a customizable approach that supports patients throughout their care journey, from diagnosis to long-term follow-up. The key advantages include access to scientifically validated information, flexibility to learn at one’s own pace, and the opportunity to revisit content as needed. Remaining questions concern the long-term impact of such programs on clinical outcomes, adherence, and quality of life. Beyond improving knowledge and engagement, this MOOC fosters patient self-management and strengthens the partnership between patients and health care professionals. Its success underscores the importance of co-designing educational resources with patients and highlights the potential of digital platforms to advance chronic disease care in a sustainable, equitable, and patient-centered manner. The program was highly valued by users and demonstrates the feasibility of large-scale digital patient education in oncology. Its design and outcomes may serve as a model for future digital health education initiatives targeting other chronic diseases.

## Abbreviations

AF3M: Association Française des Malades du Myélome Multiple (French Association of Patients with Multiple Myeloma)
ANSM: French National Agency for Medicines and Health Products Safety
IFM: Intergroupe Francophone du Myélome (French-Speaking Myeloma Intergroup)
MM: multiple myeloma
MOOC: massive open online course
RIPH: Research Involving the Human Person
TPE: therapeutic patient education

## References

[1] Smith-Lickess SK, Woodhead T, Burhouse A, Vasilakis C (2019). Study design and protocol for a comprehensive evaluation of a UK massive open online course (MOOC) on quality improvement in healthcare. BMJ Open.

[2] Carvalho PF, Sana F, Yan VX (2020). Self-regulated spacing in a massive open online course is related to better learning. NPJ Sci Learn.

[3] King C, Robinson A, Vickers J (2014). Online education: targeted MOOC captivates students. Nature New Biol.

[4] Berman AH, Biguet G, Stathakarou N (2017). Virtual patients in a behavioral medicine massive open online course (MOOC): a qualitative and quantitative analysis of participants’ perceptions. Acad Psychiatry.

[5] Hossain MS, Shofiqul Islam M, Glinsky JV, Lowe R, Lowe T, Harvey LA (2015). A massive open online course (MOOC) can be used to teach physiotherapy students about spinal cord injuries: a randomised trial. J Physiother.

[6] Gardair C, Bousquet G, de Bazelaire C (2017). Massive open online course (MOOC) sur le diagnostic des cancers: bilan et évaluation de l’impact sur la perception de l’anatomie et cytologie pathologiques. Ann Pathol.

[7] Pottier E, Boulanouar L, Bertrand M (2020). A MOOC about bariatric surgery improves knowledge and promotes patients’ soft skills. OBES SURG.

[8] Larsson C, Marley J, Piccinini F (2025). The MyRelief digital educational self-management program for persistent low back pain: feasibility uncontrolled trial. JMIR Form Res.

[9] Purvis T, Burns C, Barker S (2025). Co-designing a digital stroke prevention platform: leveraging lived experience and expert advice. Health Expect.

[10] Álvarez-Pérez Y, Duarte-Díaz A, Toledo-Chávarri A (2023). Digital health literacy and person-centred care: co-creation of a massive open online course for women with breast cancer. Int J Environ Res Public Health.

[11] Nelligan RK, Hinman RS, Egerton T (2023). Effects of a massive open online course on osteoarthritis knowledge and pain self-efficacy in people with hip and/or knee osteoarthritis: protocol for the MOOC-OA randomised controlled trial. BMC Musculoskelet Disord.

[12] Nicolas C, Balardy L, Antoine V (2022). Spreading geriatric oncology culture through professional caregivers: results of a French massive open online course (MOOC). J Geriatr Oncol.

[13] Article R1121-1. https://www.legifrance.gouv.fr/.

[14] Alturkistani A, Lam C, Foley K (2020). Massive open online course evaluation methods: systematic review. J Med Internet Res.

